# Severe osteoporosis in a young man with bilateral Cushing’s syndrome: a case report

**DOI:** 10.1186/s13256-023-03968-0

**Published:** 2023-06-17

**Authors:** Bárbara Oliveira Reis, Christianne Toledo Sousa Leal, Danielle Guedes Andrade Ezequiel, Ana Carmen dos Santos Ribeiro Simões Juliano, Flávia Lopes de Macedo Veloso, Leila Marcia da Silva, Lize Vargas Ferreira, Mariana Ferreira, Gabriel Zeferino De Oliveira Souza

**Affiliations:** Serviço de Endocrinologia, Hospital Universitário da Universidade Federal de Juiz de Fora, Juiz de Fora, Minas Gerais Brazil

**Keywords:** Cushing syndrome, Osteoporosis, Adrenal gland, Hypertension, Case report

## Abstract

**Background:**

The diagnosis of Cushing’s syndrome is challenging; however, through the clinical picture and the search for secondary causes of osteoporosis, it was possible to reach the diagnosis of the case reported. There was an independent, symptomatic ACTH hypercortisolism manifested by typical phenotypic changes, severe secondary osteoporosis and arterial hypertension in a young patient.

**Case presentation:**

A 20-year-old Brazilian man with low back pain for 8 months. Radiographs showed fragility fractures in the thoracolumbar spine, and bone densitometry showed osteoporosis, especially when evaluating the *Z* Score (− 5.6 in the lumbar spine). On physical examination, there were wide violaceous streaks on the upper limbs and abdomen, plethora and fat increase in the temporal facial region, hump, ecchymosis on limbs, hypotrophy of arms and thighs, central obesity and kyphoscoliosis. His blood pressure was 150 × 90 mmHg. Cortisol after 1 mg of dexamethasone (24.1 µg/dL) and after Liddle 1 (28 µg/dL) were not suppressed, despite normal cortisoluria. Tomography showed bilateral adrenal nodules with more severe characteristics. Unfortunately, through the catheterization of adrenal veins, it was not possible to differentiate the nodules due to the achievement of cortisol levels that exceeded the upper limit of the dilution method. Among the hypotheses for the differential diagnosis of bilateral adrenal hyperplasia are primary bilateral macronodular adrenal hyperplasia, McCune–Albright syndrome and isolated bilateral primary pigmented nodular hyperplasia or associated with Carney’s complex. In this case, primary pigmented nodular hyperplasia or carcinoma became important etiological hypotheses when comparing the epidemiology in a young man and the clinical-laboratory-imaging findings of the differential diagnoses. After 6 months of drug inhibition of steroidogenesis, blood pressure control and anti-osteoporotic therapy, the levels and deleterious metabolic effects of hypercortisolism, which could also impair adrenalectomy in the short and long term, were reduced. Left adrenalectomy was chosen, given the possibility of malignancy in a young patient and to avoid unnecessary definitive surgical adrenal insufficiency if the adrenalectomy was bilateral. Anatomopathology of the left gland revealed expansion of the zona fasciculate with multiple nonencapsulated nodules.

**Conclusion:**

The early identification of Cushing’s syndrome, with measures based on the assessment of risks and benefits, remains the best way to prevent its progression and reduce the morbidity of the condition. Despite the unavailability of genetic analysis for a precise etiological definition, it is possible to take efficient measures to avoid future damage.

**Supplementary Information:**

The online version contains supplementary material available at 10.1186/s13256-023-03968-0.

## Background

Cushing’s syndrome may be exogenous or endogenous and, in this case, can be ACTH-dependent or independent. In the case reported, there was an independent, symptomatic ACTH hypercortisolism manifested by typical phenotypic changes, severe secondary osteoporosis and arterial hypertension in a young patient. Osteoporosis secondary to hypercortisolism occurs due to chronic reduction in bone formation, loss of osteocytes and increased reabsorption caused by intense binding of cortisol to glucocorticoid receptors present in bone cells [1]. In addition, excess cortisol impairs vitamin D metabolism and reduces endogenous parathyroid hormone secretion, intestinal calcium reabsorption, growth hormone release, and lean body mass [2]. Subclinical Cushing disease occurs in up to 11% of individuals diagnosed with early-onset osteoporosis and 0.5–1% of hypertension patients^.^ [3] A cross-sectional study published in 2023 revealed a prevalence of 81.5% bone loss in 19 patients with Cushing’s syndrome [2]. The prevalence of osteopenia ranges from 60 to 80%, and the prevalence of osteoporosis ranges from 30 to 65% in patients with Cushing’s syndrome. Additionally, the incidence of fragility fractures ranges from 30 to 50% in these patients [4] and is considered the main cause of morbidity affecting the quality of life. The diagnosis is challenging, given the presence of confounding factors; however, through the clinical picture and the search for secondary causes of osteoporosis, it was possible to reach a syndromic diagnosis. Early identification of this syndrome, with measures based on the assessment of risks and benefits, remains the best way to prevent progression and reduce morbidity related to this disease [2].

## Case presentation

A 20-year-old Brazilian male patient reported low back pain that had evolved for 8 months, with no related trauma. He sought emergency care and performed spinal radiographs on this occasion (03/2019). Due to the several alterations observed in the images, he was referred to the Orthopedics Service of the Hospital of Federal University of Juiz de Fora, which prescribed orthopedic braces, indicated physical therapy and was referred again to the Osteometabolic Diseases outpatient clinic of the Endocrinology and Rheumatology Services of the Hospital of Federal University of Juiz de Fora on 10/2019.

**Fig. 1:**
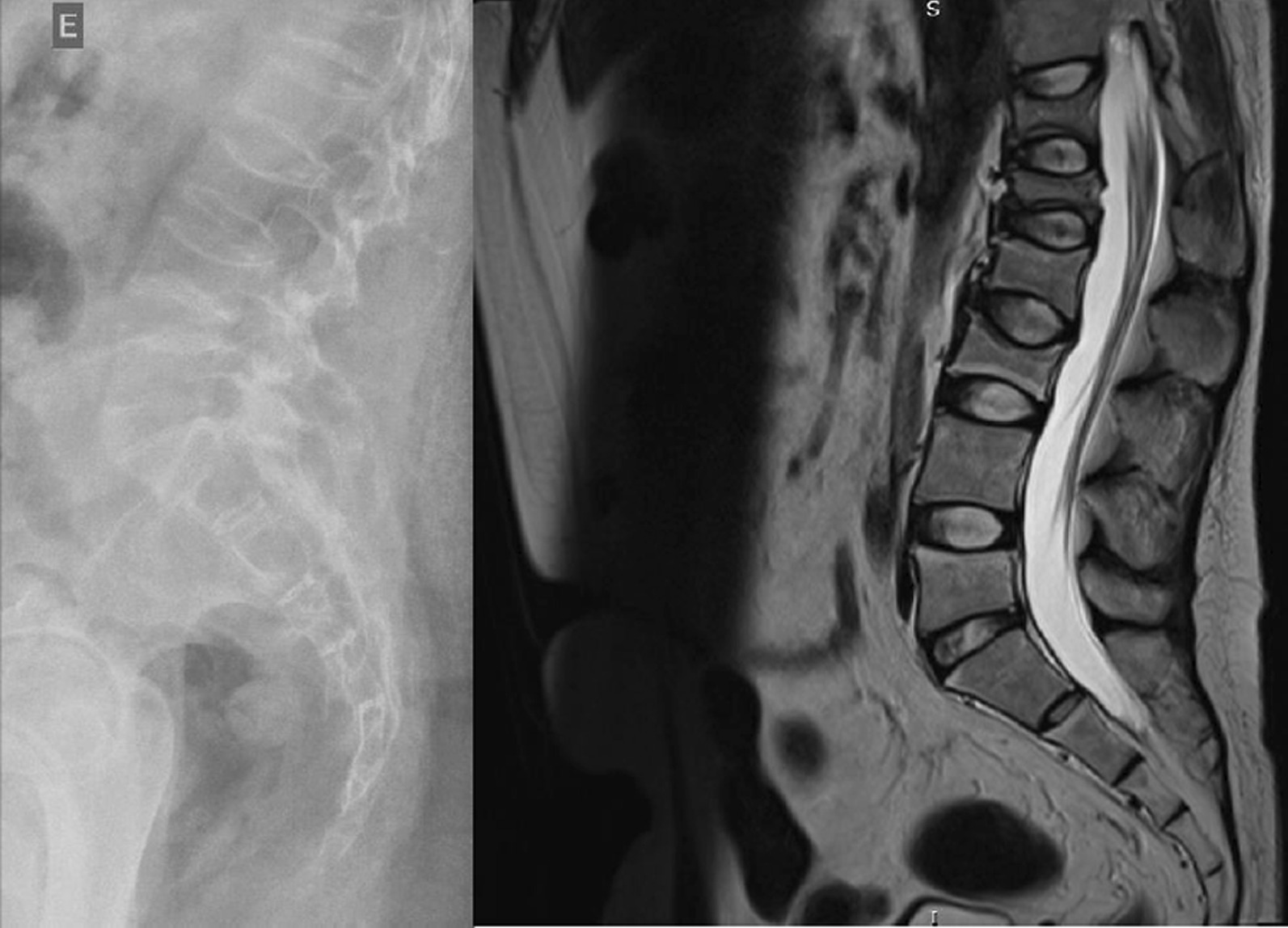
Radiography and Magnetic Resonance Imaging (MRI) of lumbosacral spine in profile

The radiographs showed a marked reduction in the density of bone structures, scoliotic deviation with convexity toward the left and reduction in the height of the lumbar vertebrae, with partial collapses of the vertebral bodies at the level of T12, L1, L2, L3 and L5, with recent collapses in T12 and L1, suggesting bone fragility fractures. The same can be seen in posterior magnetic resonance imaging (Fig. 1).

Bone scintigraphy on 08/2019 did not reveal hyper flow or anomalous hyperemia in the topography of the thoracolumbar spine, and in the later images of the exam, there was a greater relative uptake of the tracer in the lumbar spine (vertebrae T10–T12, L2–L4), of nonspecific aspect, questioning the presence of osteoarticular processes or ankylosing spondylitis.

It was also observed in the bone densitometry requested in October 2019, performed by dual-energy X-ray absorptiometry (DXA), low bone mineral density (BMD) in the lumbar spine, femoral neck and total femur, when comparing the results to evaluating the *Z* Score (Table 1).

**Table 1:** Dual-energy X-ray absorptiometry (DXA)

Analyzed site	DMO (g/cm^2^)	*Z* score	*T* score
L2–L4 (L3)	0.570	− 5.6	− 5.6
Neck	0.610	− 3.8	− 3.5
Total	0.678	− 3.0	− 2.9

Thus, the diagnosis of osteoporosis was established, and treatment with vitamin D 7000 IU per week was started due to vitamin D3 insufficiency associated with the bisphosphonate alendronate 70 mg, also weekly. The patient had a past pathological history of fully treated syphilis (2018) and perianal condyloma with a surgical resection on 09/2017 and 02/2018. In the family history, it was reported that a maternal uncle died of systemic sclerosis. In the social context, the young person denied drinking alcohol and previous or current smoking.

**Fig. 2:**
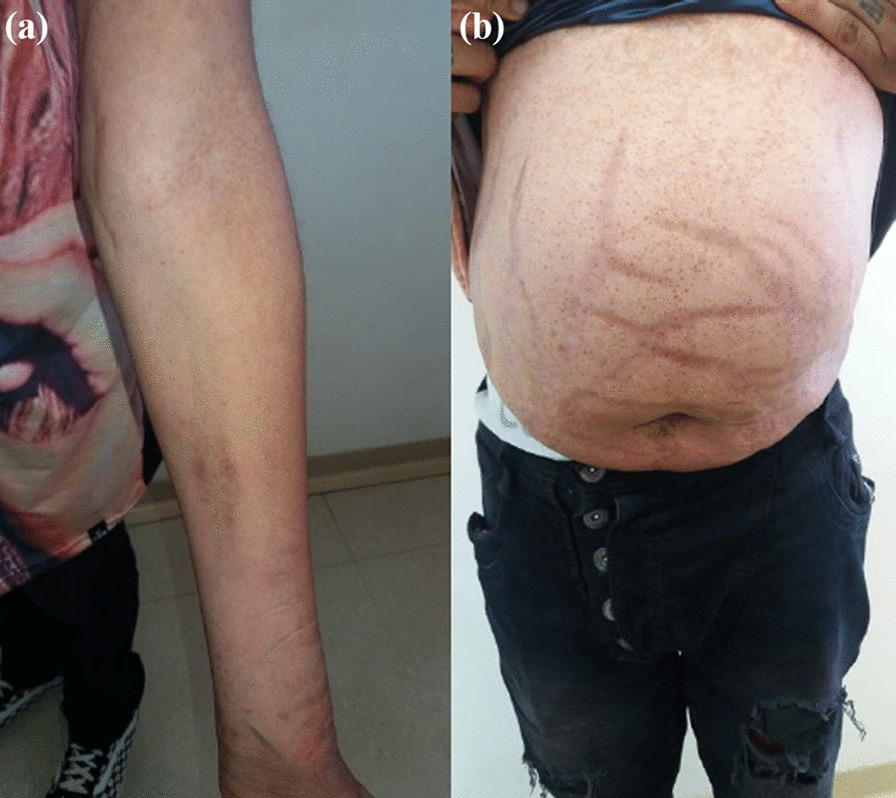
Changes in the physical examination. **a** Violet streaks on the upper limbs, **b** Violet streaks on abdomen

On physical examination, there were no lentiginous skin areas or blue nevi; however, wide violet streaks were observed on the upper limbs and abdomen, with plethora and increased fat in the temporal facial region and hump (Fig. 2a, b), limb ecchymosis, hypotrophy of the arms and thighs, central obesity and kyphoscoliosis. Systemic blood pressure (sitting) was 150 × 90 mmHg, BMI was 26.09 kg/m^2^, and waist circumference was 99 cm, with no reported reduction in height, maintained at 1.55 m.

An investigation of secondary causes for osteoporosis was initiated, with the following laboratory test results (Table 2).

**Table 2:** Laboratory tests

Exam	Result	Reference value
Hematocrit	41.4%	(41–54)
Leukocytes	9684 μL	(3600–11,000)
Platelets	172,600 μL	(150,000–400,000)
Creatinine	0.7 mg/dL (MDRD 143.78 mL/min/1.73 m^2^)	(0.7–1.2)
Alkaline phosphatase	485 U/L	(65–300)
Pyruvic transaminase	20 U/L	(< 41)
Oxalacetic transaminase	14 U/L	(< 38)
Albumin	4.3 g/dL	(3.5–4.8)
Total calcium	10.3 mg/dL	(8.5–10.5)
Potassium	4.2 mEq/L	(3.6–5.5)
Sodium	141 mEq/L	(134–149)
Phosphor	2.5 mg/dL	(2.5–7.0)
Magnesium	1.9 mg/dL	(1.6–2.6)
Parathyroid hormone	61.7 pg/mL	(15.0–68.3)
D vitamin	21.8 ng/mL	(> 20)
Anti-HIV	Nonreactive	Nonreactive
Fasting blood glucose	78 mg/dL	(65–99)
Glycated hemoglobin	5.3%	(4.8–5.9)
Total cholesterol	222 mg/dL	(< 200)
LDL cholesterol	151 mg/dL	(< 100)
HDL cholesterol	51 mg/dL	(> 60)
Triglycerides	99 mg/dL	(< 150)
Erythrocyte sedimentation rate in the 1st hour	17 mm	(0–10)
C-reactive protein	1 mg/L	(< 6)
Immunoglobulin A	137 mg/dL	(70–400)
Iga anti-transglutaminase	1.1 U/mL	(< 7,0)
Basal cortisol	26.2 μg/dL	(3.7–19.4)
Cortisol after 1 mg of dexamethasone	24.1 μg/dL	(< 1.0)
Cortisol after liddle 1	28 μg/dL	(< 1.0)
Corticotropin (in two doses)	< 5.0 pg/mL	(< 46)
Thyrotropin	1.2692 uUI/mL	(0.35–4.9)
Free thyroxine	0.88 ng/dL	(0.7–1.48)
Prolactin	11.51 ng/mL	(3.5–19.4)
Total testosterone	1.47 ng/mL	(1.42–9.23)
Abnormal elements of urinary sediment	With amorphous phosphate crystals++	Absent
Calciuria	213 mg/24 h	(100–300)
Phosphaturia	240 mg/24 h	(340–1000)
Cortisoluria	60.3 μg/24 h	(4.3–176.07)

(%) percentage, (uL) microliter, (mg) milligram, (dL) deciliter, (MDRD) Modification of Diet in Renal Disease Study Group equation, (U) measurement Units, (g) grams, (L) liter, (mEq) thousand equivalent, (pg) picogram, (ng) nanogram, (uUI) micro measurement Units, (h) hours, (HIV) human immunodeficiency virus, (LDL) low-density lipoprotein, (HDL) high-density lipoprotein

**Fig. 3:**
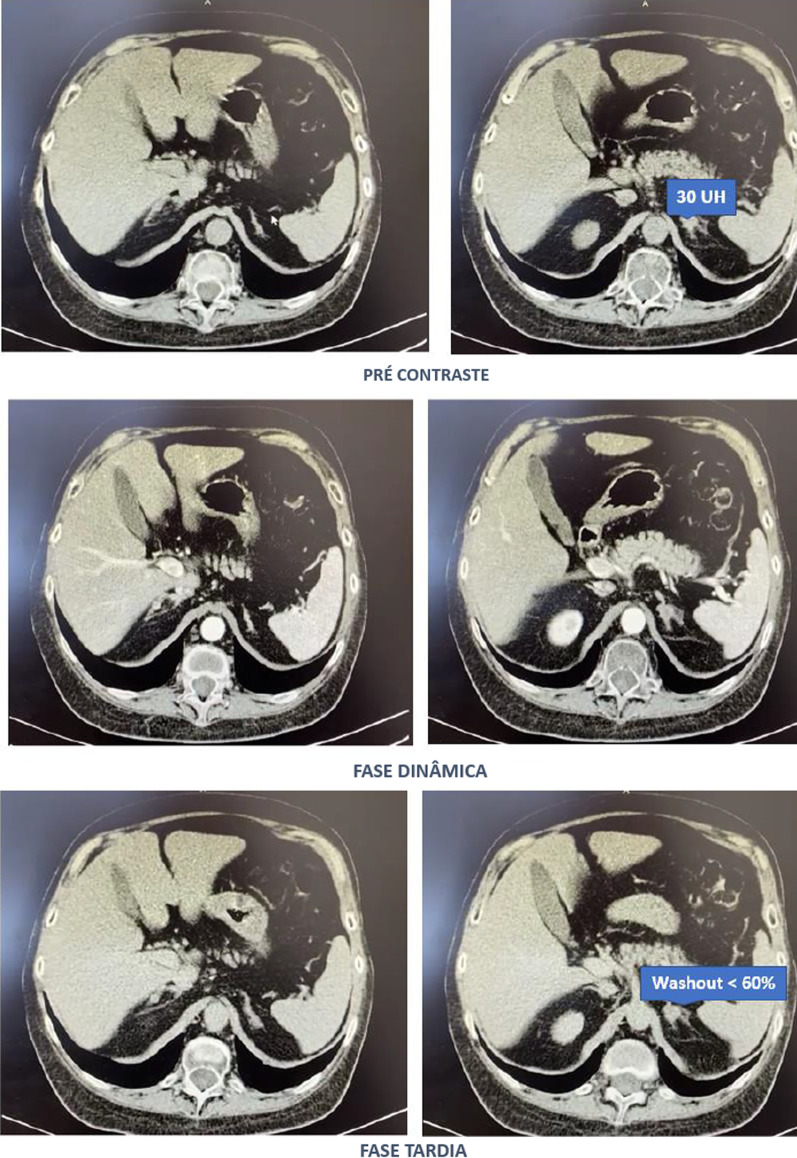
Computed tomography abdomen with adrenal protocol

Computed tomography of the abdomen with adrenal protocol performed on 08/13/2020 characterized isodense nodular formation in the body of the left adrenal and in the lateral portion of the right adrenal, measuring 1.5 cm and 0.6 cm, respectively. The lesions had attenuation of approximately 30 HU, showing enhancement by intravenous contrast, with an indeterminate washout pattern in the late phase after contrast (< 60%) (Fig. 3).

After contact with the interventional radiology of the Hospital of Federal University of Juiz de Fora, catheterization of adrenal veins was performed on 10/2020; however, it was not possible to perform adequate lesion characterization due to obtaining serum cortisol levels that extrapolated the dilutional upper limit of the method (Table 3).

**Table 3:** Adrenal catheterization

Vase	Cortisol	Aldosterone	Dehydroepiandrosterone sulfate
Peripheral vein	22.6 mcg/dL	Not collected	Not collected
Adrenal veins	150 and 150 mcg/dL (sample dilution 1:2)	5.4 and 6.1 ng/dL (RV: 1.8 to 23.2)	113 and 128 mcg/dL (RV: 85–690)

The calculation of the selectivity index was 6.63 (Reference Value (RV) > 3), confirming the good positioning of the catheter within the vessels during the procedure. The calculated lateralization index was 1.1296 (VR < 3), denoting bilateral hormone production. However, as aldosterone was not collected from a peripheral vein, it was not possible to obtain the contralateral rate and define whether there was contralateral suppression of aldosterone production [5].

Due to pending diagnoses for a better therapeutic decision and Cushing’s syndrome in clear evolution and causing organic damage, it was decided, after catheterization, to make changes in the patient’s drug prescription. Ketoconazole 400 mg per day was started, the dose of vitamin D was increased to 14,000 IU per week, and ramipril 5 mg per day was prescribed due to secondary hypertension. In addition, given the severity of osteoporosis, it was decided to replace previously prescribed alendronate with zoledronic acid.

Magnetic resonance imaging of the upper abdomen was performed on 06/19/2021, which demonstrated lobulated nodular thickening in the left adrenal gland with areas of decreased signal intensity in the T1 out-phase sequence, denoting the presence of fat, and homogeneous enhancement using contrast, measuring approximately 1.7 × 1.5 × 1.3 cm, suggestive of an adenoma. There was also a small nodular thickening in the lateral arm of the right adrenal, measuring approximately 0.8 × 0.6 cm, which was difficult to characterize due to its small dimensions and nonspecific appearance.

PPNAD or carcinoma became an important etiological hypothesis for the case described when comparing the epidemiology in a young man and the clinical-laboratory-imaging findings of the differential diagnoses. According to a dialog with the patient and family, the group of experts opted for unilateral glandular surgical resection on the left side (11/11/2021), where more significant changes were visualized, as there was a possibility of malignancy in a young patient and to avoid a definitive adrenal insufficiency condition because of bilateral adrenalectomy. This would first allow the analysis of the material and follow-up of the evolution of the condition with the permanence of the contralateral gland.

**Fig. 4:**
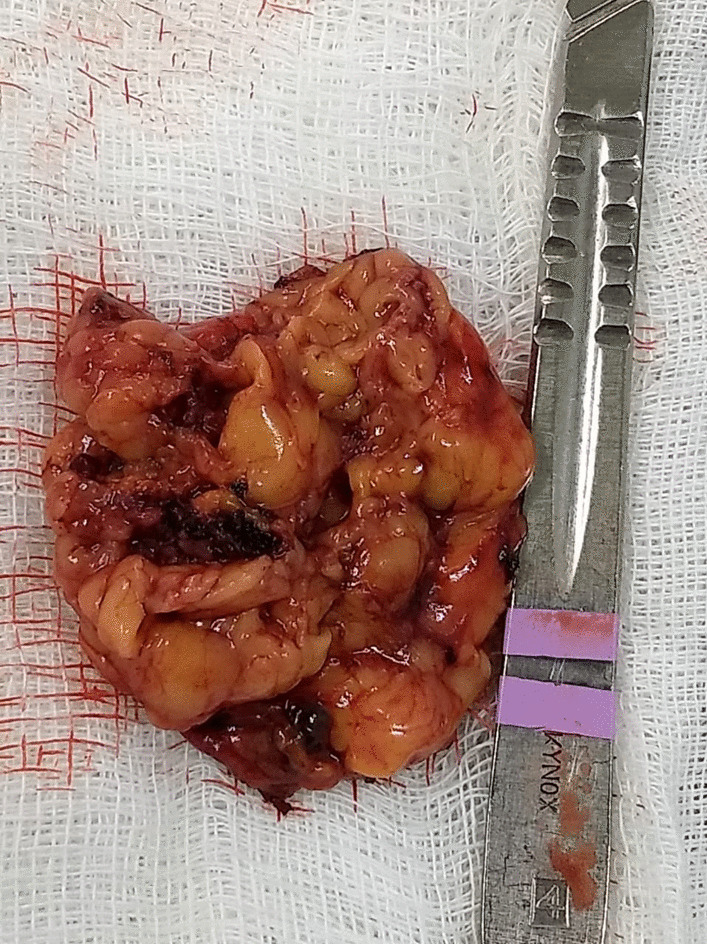
Left adrenal

In the macroscopic analysis of the adrenalectomy specimen, adrenal tissue weighing 20 g and measuring 9.3 × 5.5 × 2.0 cm was described, completely surrounded by adipose tissue. The gland has a multinodular surface and varies between 0.2 and 1.6 cm in thickness, showing a cortex of 0.1 cm in thickness and a medulla of 1.5 cm in thickness (Fig. 4).

The microscopic analysis described the expansion of the zona fasciculate, with the formation of multiple nonencapsulated nodules composed of polygonal cells with ample and eosinophilic cytoplasm and frequent depletion of intracytoplasmic lipid content. No areas of necrosis or mitotic activity were observed. The histopathological picture is suggestive of cortical pigmented micronodular hyperplasia of the adrenal gland.

For the final etiological definition and an indication of contralateral adrenalectomy, which could be unnecessary and would avoid chronic corticosteroid therapy, or else, it would be necessary to protect the patient from future complications with the maintenance of the disease in the right adrenal gland, it would be essential to search for mutations in the PRKAR1A, PDE11A, PDE8B and PRKACA genes [15]; however, such genetic analysis is not yet widely available, and the impossibility of carrying it out at the local level did not allow a complete conclusion of the case.

## Discussion

Through the clinical picture presented and the research of several secondary causes for osteoporosis, it was possible to arrive at the diagnosis of Cushing syndrome [6]. There was symptomatic independent ACTH hypercortisolism, manifested by typical phenotypic changes, severe secondary osteoporosis, and arterial hypertension in a young patient.

The diagnosis of Cushing’s syndrome is always challenging, given the presence of confounding factors such as the following:

- Physiological states of hypercortisolism—pseudo Cushing (strenuous exercise, pregnancy, uncontrolled diabetes, sleep apnea, chronic pain, alcohol withdrawal, psychiatric disorders, stress, obesity, glucocorticoid resistance syndromes);
- Cyclic or mild—subclinical Cushing’s pictures;
- Frequent and, even unknown, short- and long-term use of corticosteroids under different presentations;
- Increase in the general population incidence of diabetes and obesity;
- Screening tests with singularities for collection and individualized for different patient profiles.

It is important to note that the basal morning cortisol measurement is not the ideal test to assess hypercortisolism and is better applied to the assessment of adrenal insufficiency. However, the hypercortisolism of the case was unequivocal, and this test was also shown to be altered several times. As no test is 100% accurate, the current guidelines suggest the use of at least two first-line functional tests that focus on different aspects of the pathophysiology of the hypothalamic‒pituitary‒adrenal axis to confirm the hypercortisolism state: 24-hours cortisol, nocturnal salivary cortisol, morning serum cortisol after suppression with 1 mg of dexamethasone or after Liddle 1. Given that night-time salivary cortisol would require hospitalization, the other suggested tests were chosen, which are easier to perform in this context [7, 8].

Subsequently, tests were performed to determine the cause of hypercortisolism, such as serum ACTH levels and adrenal CT. The suppressed ACTH denoted the independence of its action. CT showed bilateral adrenal nodules with more severe features: solid lesion, attenuation > 10 UI on noncontrast images, and contrast washout speed < 60% in 10 minutes. In this case, it is essential to make a broad clinical decision and dialog with the patient to weigh and understand the risks and benefits of surgical treatment [9].

Among the main diagnostic hypotheses for the differential diagnosis of bilateral adrenal hyperplasia are primary bilateral macronodular adrenal hyperplasia, McCune–Albright syndrome (MAS) and bilateral primary pigmented nodular hyperplasia (PPNAD) isolated or associated with Carney’s complex. Another possibility would be bilateral adrenocorticotropic hormone (ACTH)-dependent macronodular hyperplasia secondary to long-term adrenal stimulation in patients with Cushing’s disease (ACTH-secreting pituitary tumor) or ectopic ACTH production, but the present case did not present with ACTH elevation.

Primary macronodular adrenal hyperplasia (nodules > 1 cm) predominates in women aged 50–60 years and may also be detected in early childhood (before 5 years) in the context of McCune–Albright syndrome. Most cases are considered sporadic; however, there are now several reports of familial cases whose presentation suggests autosomal dominant transmission. Several pathogenic molecular causes were identified in the table, indicating that it is a heterogeneous disease [10]. The pathophysiology occurs through the expression of anomalous ectopic hormone receptors or amplified eutopic receptors in the adrenals. It usually manifests in an insidious and subclinical way, with cortisol secretion mediated through receptors for gastric inhibitory peptide (GIP), vasopressin (ADH), catecholamines, interleukin 1 (IL-1), leptin, luteinizing hormone (LH), serotonin or others. Nodular development is not always synchronous or multiple; thus, hypercortisolism only manifests when there is a considerable increase in the number of adrenocortical cells, with severe steroidogenesis observed by cortisoluria greater than 3 times the upper limit of normal. Patients with mild Cushing’s syndrome should undergo screening protocols to identify aberrant receptors, as this may alter the therapeutic strategy. If there is evidence of abnormal receptors, treatment with beta-blockers is suggested for patients with beta-adrenergic receptors or with gonadotropin-releasing hormone (GnRH) agonists (and sex steroid replacement) for patients with LH/hCG receptors. In patients in whom aberrant hormone receptors are not present or for whom no specific pharmacological blockade is available or effective, the definitive treatment is bilateral adrenalectomy, which is known to make the patient dependent on chronic corticosteroid therapy [11]. Studies have shown the effectiveness of unilateral surgery in the medium and long term, opting for the resection of the adrenal gland of greater volume and nodularity by CT, regardless of the values obtained by catheterization of adrenal veins, but with the possibility of persistence or recurrence in the contralateral gland. Another possibility would be total unilateral adrenalectomy associated with subtotal contralateral adrenalectomy [12].

In McCune–Albright syndrome (MAS), there are activating mutations in the G-protein GNAS1 gene, generating autonomic hyperfunction of several tissues, endocrine or not, and there may be, for example, a constant stimulus similar to ACTH on the adrenal gland. In this case, pituitary levels of ACTH are suppressed, and adrenal adenomas with Cushing’s syndrome appear. Hypercortisolism may occur as an isolated manifestation of the syndrome or be associated with the triad composed of polyostotic fibrous dysplasia, café au lait spots with irregular borders and gonadal hyperfunction with peripheral precocious puberty. The natural history of Cushing’s syndrome in McCune-Albright syndrome (MAS) is heterogeneous, with some children evolving with spontaneous resolution of hypercortisolism, while others have a more severe condition, eventually requiring bilateral adrenalectomy [13].

PPNAD predominates in females, in people younger than 30 years, multiple and small (< 6 mm) bilateral pigmented nodules (surrounded by atrophied cortex), which can reach 1.5 cm in adulthood, with family genetic inheritance (66%) or sporadic inheritance (33%), and as part of the Carney complex reported in 40% of cases. In 70% of cases, inactivating mutations are identified in the PKA regulatory 1-alpha subunit (PRKAR1A), a tumor suppressor gene [14]. Osteoporosis is often associated with this condition [15]. One test that can distinguish patients with PPNAD from other primary adrenocortical lesions is cortisoluria after sequential suppression with low- and high-dose dexamethasone. In contrast to most patients with primary adrenocortical disease, who demonstrate no change in urinary cortisol, 70% of PPNAD patients have a paradoxical increase in urinary cortisol excretion [16]. The treatment of choice for PPNAD is bilateral adrenalectomy due to the high recurrence rate for primary adrenal disease [17].

Carney complex is a multiple neoplastic syndrome with autosomal dominant transmission, characterized by freckle-like cutaneous hyperpigmentation (lentiginosis), endocrine tumors [(PPNAD), testicular and/or thyroid tumors and acromegaly] and nonendocrine tumors, including cutaneous, cardiac, mammary, and osteochondral myxomas, among others. In the above case, the transthoracic echocardiogram of the patient on 03/18/2021 showed cavities of normal dimensions, preserved systolic and diastolic functions, no valve changes and no lentiginous skin areas and blue nevi, making the diagnosis of the syndrome less likely. The definitive diagnosis of Carney requires two or more main manifestations. Several related clinical components may suggest the diagnosis but not define it. The diagnosis can also be made if a key criterion is present and a first-degree relative has Carney or an inactivating mutation of the gene encoding PRKAR1A [18].

The adenoma is usually small in size (< 3 cm), similar to the nodules in this case; however, it is usually unilateral, with an insidious and mild evolution, especially in adult women over 35 years of age, producing only 1 steroid class. Carcinomas are usually large (> 6 cm), and only 10% are bilateral. They should be suspected mainly when the tumor presents with hypercortisolism associated with hyperandrogenism. They have a bimodal age distribution, with peaks in childhood and adolescence, as well as at the end of life [3].

## Conclusion

Early identification of Cushing’s syndrome, with measures based on the assessment of risks and benefits, remains the best way to prevent progression and reduce morbidity [2]. After 6 months of drug inhibition of steroidogenesis, blood pressure control and anti-osteoporotic therapy, the objective was to minimize the levels and deleterious metabolic effects of hypercortisolism, which could also harm the surgical procedure in the short and long term through infections, dehiscence, nonimmediate bed mobilization and cardiovascular events. Unilateral adrenalectomy was chosen, given the possibility of malignancy in a young patient and to avoid definitive surgical adrenal insufficiency if the adrenalectomy was bilateral. Despite the unavailability of genetic analysis for a precise etiological definition, it is possible to take efficient measures to avoid unnecessary consequences or damage.

## Supplementary Information

**Additional file 1.** Surgical removal of adrenal gland.

## Data Availability

All data generated or analysed during this study are included in this published article [and its Additional file 1]. The datasets generated and/or analysed during the current study are available in the link https://ufjfedubr-my.sharepoint.com/:v:/g/personal/barbara_reis_ufjf_edu_br/EVpIR005sPZGlQvMJhIwSaUB0Hig4KOjhkG4D4cMggUwHA?e=Dk8tng.

## Abbreviations

ACTH: Adrenocorticotropic hormone
PPNAD: Bilateral primary pigmented nodular hyperplasia
DXA: Dual energy X-ray absorptiometry
GIP: Gastric inhibitory peptide
GnRH: Gonadotropin-releasing hormone
IL-1: Interleukin 1
BMD: Low bone mineral density
LH: Luteinizing hormone
MAS: McCune–Albright syndrome
PRKAR1A: PKA regulatory 1-alpha subunit
ADH: Vasopressin
